# Efficacy of oral afoxolaner against *Amblyomma maculatum* infestations in dogs

**DOI:** 10.1051/parasite/2025032

**Published:** 2025-06-24

**Authors:** Eric Tielemans, Pascal Dumont, Carin Rautenbach, Alta Viljoen, Joseph Prullage

**Affiliations:** 1 Boehringer Ingelheim Animal Health 29 avenue Tony Garnier 69007 Lyon France; 2 Boehringer Ingelheim Animal Health USA Inc. 1730 Olympic Drive Athens GA 30601 USA; 3 Clinvet International (Pty) Ltd. P.O. Box 11186, Universitas Bloemfontein 9321 Republic of South Africa; 4 Boehringer Ingelheim Animal Health USA Inc., Missouri Research Center 6498 Jade Rd. Fulton MO 65251 USA

**Keywords:** Afoxolaner, *Amblyomma maculatum*, Dog

## Abstract

*Amblyomma maculatum*, the Gulf Coast tick, is a species of significant veterinary and public health importance, especially because it is a vector of important diseases, such as American canine hepatozoonosis and tidewater spotted fever. *Amblyomma maculatum* infests a wide range of vertebrates including livestock, dogs, cats, and humans. Two experimental studies were conducted to evaluate the efficacy of afoxolaner formulated in an oral tablet (NexGard^®^) against induced infestations of *A. maculatum* in dogs. These Good Clinical Practice (GCP) studies used a randomized, negative controlled and masked design. In each study, 10 dogs were allocated to an untreated group and 10 dogs to a treated group, dosed once on Day 0 with a combination of tablets targeting the minimum therapeutic dose (2.5 mg/kg afoxolaner). Dogs were infested with 50 unfed adult *A. maculatum* on Days −2, 7, 14, 21, 28, and 35 (Study #1), or on Days −1, 14, and 28 (Study #2). Seventy-two (72) hours after treatment and subsequent infestations, ticks were removed and the numbers of live ticks in each group were used for efficacy calculations. At each time-point, all untreated dogs were adequately infested (*i.e.*, with more than 12 live ticks), demonstrating a vigorous tick population and an adequate study model. The curative efficacy against established infestations, 72 hours after treatment, was 100% in Study #1 and 99.5% in Study #2. The preventive efficacy, 72 hours after the post-treatment infestations, ranged from 94.6% to 98.9% for five weeks in Study #1, and was ≥98.8% for four weeks in Study #2.

## Abstract (fr)

*Amblyomma maculatum*, la tique du Golfe du Mexique, est une espèce d’importance vétérinaire et de santé publique considérable, notamment parce qu’elle est vectrice de maladies graves, telles que l’hépatozoonose canine américaine et la fièvre pourprée des marées. *Amblyomma maculatum* infeste un large éventail de vertébrés, notamment le bétail, les chiens, les chats et les humains. Deux études expérimentales ont été menées pour évaluer l’efficacité de l’afoxolaner sous forme de comprimé oral (NexGard^®^) contre les infestations induites d’*A. maculatum* chez le chien. Ces études, conformes aux Bonnes Pratiques Cliniques (BPC), ont été randomisées, contrôlées par voie négative et en aveugle. Dans chaque étude, 10 chiens ont été répartis en un groupe non traité et 10 chiens en un groupe traité, recevant une dose unique le jour 0 d’une combinaison de comprimés ciblant la dose thérapeutique minimale (2,5 mg/kg d’afoxolaner). Les chiens ont été infestés par 50 *A. maculatum* adultes à jeun les jours −2, 7, 14, 21, 28 et 35 (étude n° 1), ou les jours −1, 14 et 28 (étude n° 2). Soixante-douze (72) heures après le traitement et les infestations ultérieures, les tiques ont été retirées et le nombre de tiques vivantes dans chaque groupe a été utilisé pour les calculs d’efficacité. À chaque point temporel, tous les chiens non traités étaient adéquatement infestés (c’est-à-dire avec plus de 12 tiques vivantes), démontrant une population de tiques vigoureuse et un modèle d’étude adéquat. L’efficacité curative contre les infestations établies, 72 heures après le traitement, était de 100% (étude n° 1) et de 99,5 % (étude n° 2). L’efficacité préventive, 72 heures après les infestations post-traitement, variait de 94,6 % à 98,9 % pendant cinq semaines dans l’étude n° 1, et était ≥ 98,8 %, pendant quatre semaines dans l’étude n° 2.

## Introduction

*Amblyomma maculatum* (Koch, 1844), also called the “Gulf Coast Tick”, is a tick species of significant veterinary and public health importance. It was originally described on the Gulf Coast of the United States (*e.g.*, Texas, South Carolina) and parts of Central and South America, mainly in livestock [2, 22]. Over the last few decades, its range has expanded to the Northeast and Midwest United States, namely in Virginia, Kansas, Oklahoma, Delaware, and New York [7, 10, 16, 19, 20, 25], likely through livestock population movement, wildlife population interactions, and through climate change [1–3, 22, 27–29]. *Amblyomma maculatum* preferably thrives in coastal lands, woodlands, and more xeric environments [6, 20, 31]. It is a three-host Ixodid tick infesting a wide range of vertebrates. Immature forms mainly feed on birds and small mammals, adults feed on larger mammals such as livestock, dogs, cats, and wildlife (*e.g.*, coyotes, panthers, and bears). In humans, *A. maculatum* infestation rates and related infectious diseases have been rising over the last few decades [22, 28, 29]. The bites of adult *A. maculatum* can cause marked and painful inflammatory reactions with consequent edema, abscesses, and predisposition to secondary infections [10, 23, 30]. Severe infestations may cause lethargy and weakness [31]. Gulf Coast ticks preferentially infest the ears of host animals. “Gotch ear” is a condition described in ruminants infested with *A. maculatum* and is characterized by external ear deformation, swelling, and drooping [8]. Importantly, *A. maculatum* is a vector of some significant human and animal pathogens. In humans, *A. maculatum* is described as the main vector of *Rickettsia parkeri*, the agent of “tidewater spotted fever”, an eschar-associated febrile illness, and of *Candidatus* Rickettsia andeanae, another spotted fever agent [13, 14, 21, 23, 30]. In animals, *A. maculatum* is the main vector of *Hepatozoon americanum,* the agent of American canine hepatozoonosis [4, 5, 9, 24]. *Hepatozoon* spp. affect a wide variety of vertebrate species, infecting the leukocytes and endothelial cells in mammalian hosts, with considerable species-dependent clinical picture diversity. American canine hepatozoonosis is a common and often severe canine disease in *A. maculatum* endemic areas, namely characterized by recurrent fever, muscle pain, and chronic muscle wasting [4, 9, 24]. The main tissue affected by *H. americanum* in dogs is the striated (vertebral and cardiac) muscle. The prognosis is often poor or guarded, with no specific treatment allowing full elimination of the parasite available. Other pathogens of importance transmitted by *A. maculatum* are *Rickettsia felis* (feline rickettsiosis), *Leptospira pomona* (livestock leptospirosis), *Ehrlichia ruminantium* (heartwater in livestock) [3, 7], and possibly *Borrelia* sp. [15].

NexGard^®^ (IVP) is an oral product for dogs containing afoxolaner, a systemic insecticide/acaricide compound belonging to the isoxazoline class. This product was designed to provide a 1-month therapeutic solution to control fleas, ticks, and mites. Its tick efficacy was namely demonstrated against *Ixodes scapularis, Rhipicephalus sanguineus, Haemaphysalis longicornis,* and *Dermacentor variabilis* [11, 12, 17, 18]. Esafoxolaner, the active enantiomer of afoxolaner has demonstrated efficacy against induced infestations of *A. maculatum* in cats [32], and afoxolaner had been tested in an unpublished preliminary study with encouraging results.

This manuscript describes two pivotal studies conducted to evaluate the efficacy of the IVP against induced infestations with *A. maculatum* in dogs.

## Materials and methods

The two single site experimental studies were conducted in 2024, Study #1 at ClinVet, Bloemfontein, Republic of South Africa, Study #2 at Boehringer Ingelheim Animal Health Inc., Athens, Georgia, USA.

### Ethics

Animals were managed with due regard for their wellbeing and the study designs were reviewed and approved by the Sponsor’s and local Institutional Animal Care and Use Committees.

### Compliance

The studies were designed in accordance with Good Clinical Practices as described in International Cooperation on Harmonization of Technical Requirements for Registration of Veterinary Medicinal Products (VICH) guideline GL9, and in accordance with the “World Association for the Advancement of Veterinary Parasitology (W.A.A.V.P.) guidelines for evaluating the efficacy of parasiticides for the treatment, prevention and control of flea and tick infestation on dogs and cats”.

### Study designs

The studies were conducted under a masked, negative controlled and randomized design. The efficacy assessments were based on comparison of live ticks found in an untreated control group and a treated group at identical time-points, 72 h after treatment and 72 h after subsequent infestations, over a period of 4–5 weeks.

### Animals, husbandry, and preliminary study activities

Twenty purpose-bred beagle dogs were used in each study. The dogs’ characteristics are summarized in Table 1. The animals were acclimated to study conditions for seven days before treatment administration, during which time they were examined and declared healthy by a veterinarian. During acclimation, dogs also underwent a preliminary *A. maculatum* infestation and count for suitability assessment and for randomization to study groups, on the basis of live tick counts in Study #1 (in Study #2 the allocation to groups was made completely at random). Dogs were individually housed in environmentally controlled indoor units. The dogs received an age-appropriate commercial dog diet and potable water was provided *ad libitum*. Dogs were observed for health daily and several times after the Day 0 treatment (1, 3 and 6 hours after treatment in Study #1, hourly for 6 h then at 8 and 12 h in Study #2).

**Table 1:** Animal characteristics.

	*n*	Breed	Bodyweight (kg) [average]	Age (months) [average]	Sex
Study #1	20	Beagle	14.6–20.0 [17.3]	13–77 [36.2]	13 m, 7 f
Study #2	20	Beagle	6.9–11.7 [9.2]	8.0–12.6 [10.2]	7 m, 13 f

### *Amblyomma maculatum* isolates

In Study #1, the *A. maculatum* ticks had originally been collected by Arachni-Med Research LLC (Sunbury, Ohio, USA) in 2021 from the field in Reno County, Kansas, USA. The colony was obtained by the test facility in March 2022 where it was maintained on rabbits not previously treated with acaricides. At least two generations were produced at the test facility before dog infestation.

In Study #2, *A. maculatum* ticks had originally been collected by Texas A&M Agrilife Research (Texas, USA), during the mid-1980s from the field in Refugio County, TX, USA. Additional progeny from gravid females collected off grazing beef cattle at the same location and from gravid females collected in another area of Texas were mixed into the colony in 1988, 1990–1991, 1999, 2007, 2014, 2018, 2019, and 2021. In 2022 and 2023, new collections of *A. maculatum* from the field were infused into the colony as part of a periodic effort to sustain the genetic variation representative of field populations.

### Treatment

Dogs were treated once on Day 0 with combinations of 0.5 g (11.3 mg afoxolaner) and 1.25 g (28.3 mg afoxolaner) NexGard^®^ tablets, calculated to provide a dose as close as possible to the minimum label dose of 2.5 mg/kg afoxolaner. In Study #1, the actual afoxolaner administered doses ranged from 2.53 to 2.81 mg/kg (average 2.67 mg/kg); in Study #2, the actual afoxolaner administered doses ranged from 2.51 to 3.05 mg/kg (average 2.76 mg/kg).

### Tick infestations and counts

Each dog was infested with 50 (±4) adult unfed *A. maculatum* (25 (±2) males and 25 (±2) females) according to the schedule described in Table 2. Dogs were infested once during acclimation for inclusion (and for randomization in Study #1), once prior to treatment to evaluate the curative efficacy of the product against established infestations, and several times after treatment to evaluate preventive/sustained efficacy. For infestations, dogs were sedated and placed in an individual crate. The ticks were placed on the back or the side of the sedated dogs for 2–4 h to ease the tick attachment process. Ticks were removed and counted 72 (±2) hours following treatment and following each subsequent infestation. For each count, ticks were identified visually or through fingertip palpation and hair parting of the whole dog body. After tick removal, the whole body was combed using a fine-toothed comb for a second check for tick presence. Each tick was observed for signs of viability or mortality. A tick was considered live when any level of motion or response to stimuli was detected. All persons participating in tick counts were masked to which treatment the dogs had received. Ticks were classified as live or dead. Protective clothing (*e.g.* gowns/coats, gloves, *etc.*) and combs were changed between each dog to prevent cross-contamination.

**Table 2:** Schedule of main study activities.

	Study #1	Study #2
Inclusion infestation	Day −7	Day −6
Inclusion count	Day −5	Day −3
Pre-treatment infestation	Day −2	Day −1
Treatment	Day 0	Day 0
Count	Day 3	Day 3
Infestation	Day 7	–
Count	Day 10	–
Infestation	Day 14	Day 14
Count	Day 17	Day 17
Infestation	Day 21	–
Count	Day 24	–
Infestation	Day 28	Day 28
Count	Day 31	Day 31
Infestation	Day 35	–
Count	Day 38	–

### Statistical analysis

The statistical unit was the individual dog. Differences in live tick counts between untreated control and treated dogs were used to determine efficacy.

The efficacy at each time-point was calculated using the formula:

Efficacy (%)=100× (LSMc – LSMt)/LSMc,

where:

LSMc = least square mean number of live ticks in the untreated control group;

LSMt = least square mean number of live ticks in the afoxolaner-treated group.

For each time point, the counts of the live ticks after treatment or subsequent infestations of each treated group were compared to the counts of the untreated control group using an F-test. A linear mixed model was used with treatment as a fixed effect and block as a random effect, at a 5% two-sided level.

The treatment was considered effective for the control of *A. maculatum*, when the following three criteria were met:

- Adequate infestation, defined as ≥ 25% (12 ticks) retention of live ticks in at least six control dogs and at each time-point.
- Calculated efficacy of ≥ 90%, at each time-point.
- Statistical comparison between the tick numbers in untreated control and treated group (with a higher number of live ticks in the control group), at each time-point, significant at the two-sided 5% level.

## Results

The efficacy results are presented in Table 3.

**Table 3:** Efficacy of afoxolaner against *Amblyomma maculatum* infestations 72 hours after treatment and subsequent weekly infestations.

	Day	Day 3	Day 10	Day 17	Day 24	Day 31	Day 38
Study #1	LSC (AM untreated control group)	36.3	35.5	40.8	35.8	32.9	35.5
	LST (AM afoxolaner treated group)	0.0	0.8	0.6	0.4	0.8	1.9
	% efficacy^1^	100.0	97.7	98.5	98.9	97.0	94.6
	*p*-value^2^	<.0001	<.0001	<.0001	<.0001	<.0001	<.0001
Study #2	AM control group	40.6	–	38.5	–	34.7	–
	AM treated group	0.2	–	0.0	–	0.4	–
	% efficacy^1^	99.5	–	100.0	–	98.8	–
	*p*-value^2^	<.0001	–	<.0001	–	<.0001	–

1 The percent reduction was calculated as 100 × [(LS_C_ − LS_T_)/LS_C_], where LS_C_ was the least square arithmetic mean of the live tick counts among the untreated control dogs and LS_T_ was the least square arithmetic mean of the live tick counts among the IVP-treated dogs.

2 *p*-value: Linear mixed model with treatment as a fixed effect and block as a random effect.

Inclusive of both studies and all efficacy evaluations, the average number of live ticks found in the untreated control groups (4 or 5 days after the initial pre-treatment infestations and 3 days after the post-treatment infestations) was 36.7 (corresponding to 73.4% retention), and individually these numbers ranged from 18 (36%) to 52 (100%). This demonstrated a vigorous tick population and adequate infestations of dogs throughout the studies with 100% of untreated dogs adequately infested at all time-points.

The curative efficacy of a single administration of afoxolaner against existing tick infestation, 72 h after treatment was 100% in Study #1 and 99.5% in Study #2. The preventive efficacy 72 h after weekly infestations over the following five weeks ranged from 94.6% to 98.9% in Study #1 and was 100% at Week 2 and 98.8% at Week 4 in Study #2.

No adverse reactions attributed to treatment with afoxolaner were observed in Study #1. One untreated dog had an ear pinnae infection caused by tick bites that required non-steroid anti-inflammatory and antibiotic treatment.

In Study #2, some instances of self-limiting diarrhea and emesis were described in comparable numbers in both the untreated and the afoxolaner treated groups; ear swelling was described for 3 untreated dogs.

## Discussion

The reduction levels of live *A. maculatum* after one IVP administration consistently exceeded 94% in the afoxolaner treated dogs for 4–5 weeks. These reduction results in the treated group were supported by the high rates of live ticks found in the untreated control animals, 72 h after treatment and after each subsequent infestation, demonstrating that the *A. maculatum* isolates used in these studies were vigorous and well adapted to canine induced infestation. Some *A. maculatum* bites caused inflammatory skin reactions in their canine host, as already described in other mammalian host species, for example in livestock [8, 22, 26], or cats in an experimental model [32]. These high attachment rates in untreated animals and local reactions to tick bites confirm that the Gulf coast tick is aggressive and should be controlled in endemic areas for animal health and welfare. Importantly, the vector role of *A. maculatum* for several human and animal diseases is a major reason for advocating its control by an acaricide product such as afoxolaner, because a reduction of adult *A. maculatum* on the canine definitive host consequently results in environmental reduction of eggs, life cycle interruptions, and an overall decrease of vector and pathogen exchanges. Most tick-borne pathogens are transmitted to their vertebrate hosts through salivary transfer within 72 h of attachment. Therefore, the experimental efficacy data produced in these studies 72 h after treatment and subsequent infestations do not provide evidence of direct protection of the canine host against tick-borne pathogens such as Rickettsiales bacteria. The situation is different with *H. americanum,* the agent of the American canine hepatozoonosis, for which *A. maculatum* is the identified vector. This protozoan is not transmitted through salivary transfer, instead it requires the host to ingest the *A. maculatum* vector containing mature oocysts. In view of the very low number of live *A. maculatum* found on treated dogs in comparison to untreated dogs in these two studies, and the mechanism of *H. americanum* transmission requiring ingestion of the vector, the hypothesis that an afoxolaner treatment may decrease the risk of transmission *via* reduced potential for ingestion of ticks carrying *H. americanum* is sound. Although to be sustained, such a hypothesis requires additional studies including vectorial infection with the protozoan.

## Conclusions

Considerable reductions of *A. maculatum* infestations in dogs treated with afoxolaner were observed in these two studies. The use of afoxolaner in *A. maculatum* endemic areas may have a beneficial effect on dog welfare and health and contribute to the control of *A. maculatum*-related vector-borne diseases.
